# Type 2 Biomarkers and Their Clinical Implications in Bronchiectasis: A Prospective Cohort Study

**DOI:** 10.1007/s00408-024-00707-0

**Published:** 2024-06-17

**Authors:** Yen-Fu Chen, Hsin-Han Hou, Ning Chien, Kai-Zen Lu, Ying-Yin Chen, Zheng-Ci Hung, Jung-Yien Chien, Hao-Chien Wang, Chong-Jen Yu

**Affiliations:** 1 Department of Internal Medicine, National Taiwan University Hospital, Yunlin Branch, Yunlin County, Taiwan; 2 Graduate Institute of Clinical Medicine, College of Medicine, National Taiwan University, Taipei, Taiwan; 3 Thoracic Medicine Center, Department of Medicine and Surgery, National Taiwan University Hospital, Yunlin Branch, Yunlin County, Taiwan; 4 Graduate Institute of Oral Biology, College of Medicine, National Taiwan University, Taipei, Taiwan; 5 Department of Medical Imaging, National Taiwan University Cancer Center, Taipei, Taiwan; 6 Department of Internal Medicine, National Taiwan University Hospital, College of Medicine, National Taiwan University, Taipei, Taiwan; 7 Precision Medicine Center, National Taiwan University Hospital, Yunlin Branch, Hu-Wei, Taiwan; 8 Department of Medicine, National Taiwan University Cancer Center, Taipei, Taiwan; 9 Department of Internal Medicine, National Taiwan University Hospital, Hsin-Chu Branch, Hsin-Chu, Taiwan

**Keywords:** Bronchiectasis, Type 2 biomarkers, Blood eosinophil count, Fractional exhaled nitric oxide, Immunoglobulin E, *Pseudomonas aeruginosa*

## Abstract

**Purpose:**

Bronchiectasis is predominantly marked by neutrophilic inflammation. The relevance of type 2 biomarkers in disease severity and exacerbation risk is poorly understood. This study explores the clinical significance of these biomarkers in bronchiectasis patients.

**Methods:**

In a cross-sectional cohort study, bronchiectasis patients, excluding those with asthma or allergic bronchopulmonary aspergillosis, underwent clinical and radiological evaluations. Bronchoalveolar lavage samples were analyzed for cytokines and microbiology. Blood eosinophil count (BEC), serum total immunoglobulin E (IgE), and fractional exhaled nitric oxide (FeNO) were measured during stable disease states. Positive type 2 biomarkers were defined by established thresholds for BEC, total IgE, and FeNO.

**Results:**

Among 130 patients, 15.3% demonstrated BEC ≥ 300 cells/μL, 26.1% showed elevated FeNO ≥ 25 ppb, and 36.9% had high serum total IgE ≥ 75 kU/L. Approximately 60% had at least one positive type 2 biomarker. The impact on clinical characteristics and disease severity was variable, highlighting BEC and FeNO as reflective of different facets of disease severity and exacerbation risk. The combination of low BEC with high FeNO appeared to indicate a lower risk of exacerbation. However, *Pseudomonas aeruginosa colonization* and a high neutrophil-to-lymphocyte ratio (NLR ≥ 3.0) were identified as more significant predictors of exacerbation frequency, independent of type 2 biomarker presence.

**Conclusions:**

Our study underscores the distinct roles of type 2 biomarkers, highlighting BEC and FeNO, in bronchiectasis for assessing disease severity and predicting exacerbation risk. It advocates for a multi-biomarker strategy, incorporating these with microbiological and clinical assessments, for comprehensive patient management.

**Supplementary Information:**

The online version contains supplementary material available at 10.1007/s00408-024-00707-0.

## Introduction

Bronchiectasis, a chronic respiratory disease, presents with cough, sputum production, dyspnea, and recurrent exacerbations often due to infections [1]. Historically, bronchiectasis was primarily linked to neutrophilic airway inflammation [2–4]. However, there has been a growing interest in understanding the role of eosinophilic inflammation in bronchiectasis [5, 6]. European cohort studies found 20% of patients have peripheral blood eosinophilia (≥ 300 eosinophils/μL) without comorbidities, termed “eosinophilic bronchiectasis” [6]. An Italian pilot study revealed a T2 high endotype—characterized by blood eosinophil counts (BEC) ≥ 300 cells/μL or fractional exhaled nitric oxide (FeNO) ≥ 25 ppb—in 31% of patients, associated with severe disease and significant quality of life impact [7]. Additionally, a Chinese study identified high serum total immunoglobulin E (IgE) (> 60 kU/L) or BEC (≥ 150 cells/µL) in 68.8% of patients, correlating with disease severity and radiological extent [8].

Recent strategies emphasize airway inflammation heterogeneity and biologics in asthma therapy [9]. This shift has led to a greater focus on evaluating composite Type 2 biomarkers, which include elevated serum IgE levels (≥ 75 kU/L), BEC (≥ 300 cells/μL), and FeNO values (≥ 25 ppb) [10]. In chronic obstructive pulmonary disease (COPD), despite less conclusive evidence, these biomarkers are instrumental for clinical outcome assessments [11–14] and in predicting responses to inhaled corticosteroids (ICS) [15] and biologics [16]. A multicenter European study in bronchiectasis linked inflammatory and microbial profiles, enhancing exacerbation prediction and biomarker development for targeted treatments [17].

Currently, the relationship between Type 2 biomarkers, clinical characteristics, disease severity, and related clinical outcomes in bronchiectasis remains poorly defined. Our study aims to explore the prevalence of positive Type 2 biomarkers and their clinical implications in East Asian individuals with bronchiectasis. To this end, we assessed three commonly used clinical markers of Type 2 inflammation: blood eosinophils, FeNO, and serum total IgE in bronchiectasis patients. Additionally, we analyzed airway microbiology and inflammation by examining bronchoalveolar lavage (BAL) samples to determine the clinical relevance of these factors in relation to disease severity and clinical outcomes.

## Material and Methods

### Study Design and Participants

Patients with bronchiectasis were prospectively enrolled from November 2018 to February 2023 at the National Taiwan University Hospital (NTUH), Yunlin Branch, Taiwan. Inclusion criteria were clinical stability and bronchiectasis diagnosis confirmed by high-resolution computed tomography (HRCT), showing a bronchoarterial ratio > 1, absence of tapering, airway visibility within 1 cm of the pleural surface [18], and symptoms like cough, dyspnea, chronic sputum production, and recurrent respiratory infections [1]. Exclusion criteria included a diagnosis of asthma or allergic bronchopulmonary aspergillosis (ABPA) [19], recent exacerbations requiring antibiotics or hospitalization within the last 3 months, or fever or acute infections in the previous 4 weeks.

At enrollment, information regarding comorbidities, past exacerbations, current inhalation medications, and exacerbation history was collected. Exacerbation severity was classified based on treatment: moderate exacerbations required outpatient antibiotics or additional therapies, while severe exacerbations necessitated hospitalization or emergency department visits for airway diseases [20]. Each patient was followed up at intervals of 3–6 months. All exacerbation outcomes, laboratory data, and imaging studies are systematically documented by the attending physicians. The thresholds for positive Type 2 biomarkers were defined as follows: BEC ≥ 300 cells/μL, serum total IgE ≥ 75 kU/L with features of atopy, and FeNO ≥ 25 ppb, in accordance with previous definitions [9, 10, 13]. The NTUH Research Ethics Committee approved this study (NTUH-REC No. 201712075RINA, 201910082RINA, and 202110093RIND).

### BAL Sample Collection

BAL sample collection and analysis were standardized. Participants fasted for 4 h before the procedure, and performed oral gargling with 20 mL 0.9% saline, followed by 0.12% chlorhexidine mouthwash. Under topical anesthesia and sedation, a bronchoscope was inserted to the selected lung region, and BAL was conducted using 200 mL of 0.9% saline [21]. The samples were processed within 2 h for analysis.

### BAL Sample for Analysis of Immune Cells and Inflammatory Cytokines

For immune and cytokine profiling, the retrieved BAL fluid was strained and cells were isolated for monoclonal antibody staining (including CD14, CD15, CD16, CD45, CD49d, CD80, CD206, CD294, CD163, CD193). Post-staining, cells underwent flow cytometry for surface antigen analysis [22]. BAL supernatants were assessed for inflammatory markers (TNF-α, IL-1β, IL-6, IL-8) with multiplex immunoassays and Luminex technology [23].

### Assessment of Severity of Bronchiectasis

Chest high-resolution computed tomography (HRCT) scans were reviewed by a trained chest specialist and a thoracic radiologist, both of whom were blinded to the clinical data. Radiological severity of bronchiectasis was quantified using a modified Reiff score, with a range from 1 (minimum) to 18 (maximum) for lobar involvement assessment [24]. The multidimensional bronchiectasis severity index (BSI) [25] and E-FACED score, encompassing exacerbation, forced expiratory volume in 1 s (FEV_1_), age, chronic colonization, disease extension, and dyspnea [26], were also utilized to comprehensively define bronchiectasis severity.

### Statistical Analysis

Continuous variables were represented by medians and interquartile range (IQR), with t-tests and Mann–Whitney tests comparing parametric and nonparametric groups, respectively. Categorical variables underwent chi-square or Fisher’s exact tests, as appropriate. For multiple group analyses, the Kruskal–Wallis test was used for continuous variables, while chi-squared or Fisher’s exact tests were used for categorical data. Spearman’s test assessed correlations between quantitative variables. Receiver operating characteristic (ROC) curves evaluated the diagnostic accuracy of clinical variables for predicting moderate to severe exacerbations. Kaplan–Meier curves depicted exacerbation-free survival, with the log-rank test for comparison. Cox’s proportional hazards model, adjusted for significant variables, was applied in the multivariable analysis of exacerbations. A two-sided p-value < 0.05 indicated statistical significance. Analyses were performed using SPSS (version 18.0, IBM).

## Results

### Prevalence and Correlation of Type 2 Biomarkers in Patients with Bronchiectasis

**Fig. 1:**
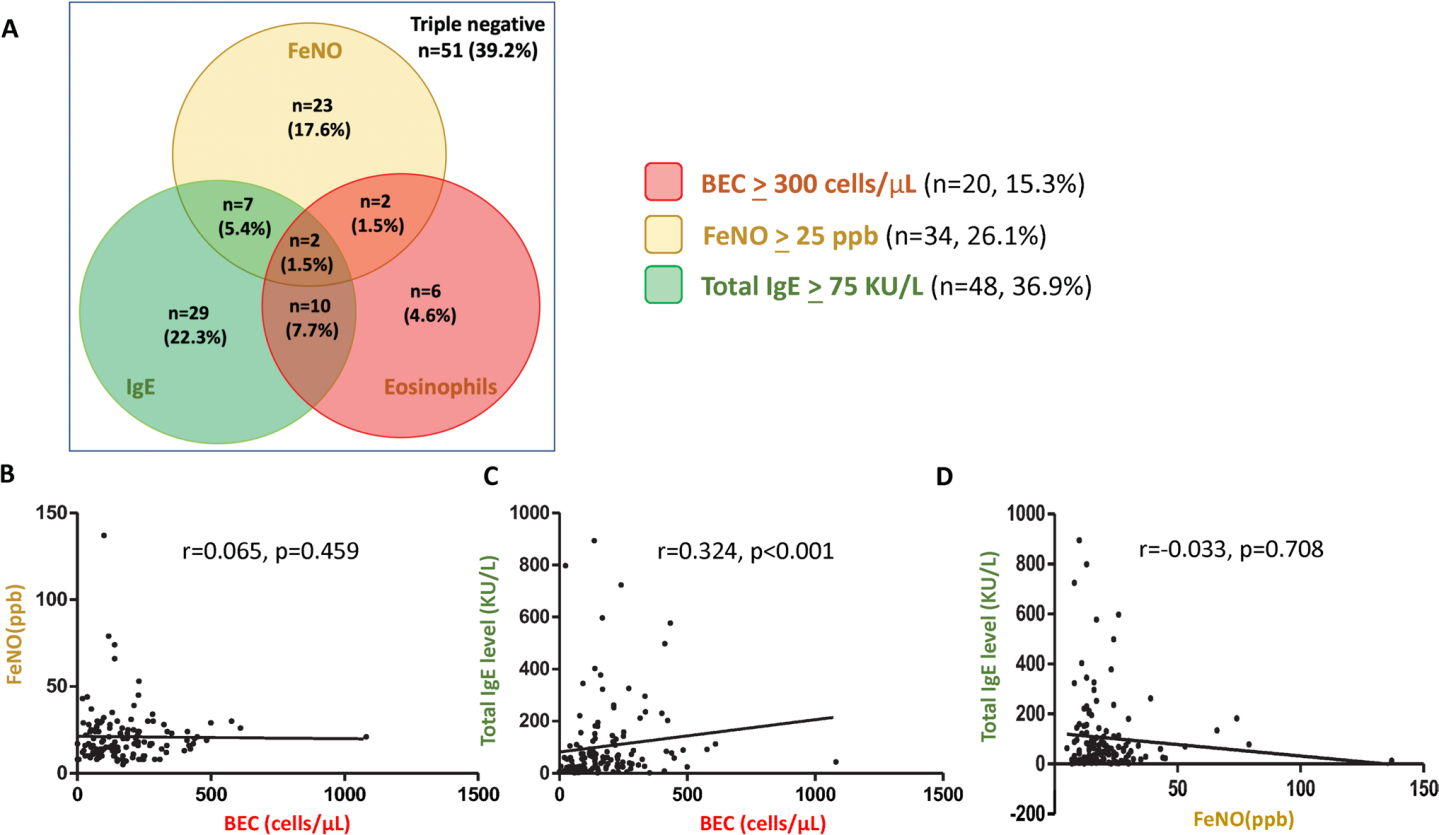
**A** overlap of baseline positivity of three clinical used biomarkers, BEC ≥ 300 cells/uL, FeNO ≥ 25 ppb, total IgE ≥ 75 kU/L in the bronchiectasis cohort (n = 130). **B**–**D** the correlation between BEC, FeNO and serum total IgE in the bronchiectasis cohort. Spearman’s test assessed correlations between quantitative variables. *BEC* blood eosinophil counts, *FeNO* fractional exhaled nitric oxide, *IgE* immunoglobulin E

A total of 145 patients with stable bronchiectasis were enrolled. Five patients could not cooperate with the bronchoscope examination, four patients had co-existing asthma, five patients lacked FeNO data, and one patient suspected of ABPA were excluded. In this analysis of 130 bronchiectasis patients, 15.3% (n = 20) had BEC ≥ 300 cells/µL, 26.1% (n = 34) exhibited FeNO levels ≥ 25 ppb, and 36.9% (n = 48) had serum total IgE levels ≥ 75 kU/L. Patients were classified into eight subgroups based on each biomarker’s positivity or negativity, as shown in Fig. 1A. Notably, 60.9% (n = 79) tested positive for at least one biomarker. A significant positive correlation was found between blood eosinophil and serum total IgE levels (r = 0.324, p < 0.001), which did not extend to their correlation with FeNO levels (r = 0.065, p = 0.459). The relationship between serum total IgE and FeNO levels was marginally negative (r = − 0.033, p = 0.708), as depicted in Fig. 1B–D.

### Clinical Profiles of Bronchiectasis Patients by Type 2 Biomarkers

Table 1 summarizes the demographics and clinical characteristics of the bronchiectasis cohort, sorted by BEC, FeNO, and IgE levels. The cohort’s median age was 67.6, predominantly male (56.9%) and nonsmoking (60.7%). Elevated BEC (≥ 300 cells/µL) was linked to a higher proportion of males (85.0%, p = 0.006), increased body mass index (BMI) (25.2, p < 0.001), and a higher percentage of smokers (65.0%, p = 0.01). The high FeNO group showed an elevated BMI (23.2, p = 0.005) but no correlation with smoking. Increased IgE levels were associated with more males (70.8%, p = 0.014) and smokers (52.1%, p = 0.022). Symptomatic and etiological profiles were consistent across biomarker stratifications. Table 2 indicates that higher IgE levels correlated with lower FEV_1_/FVC ratios (p = 0.003). BEC ≥ 300 cells/µL showed a trend towards reduced FEV_1_/FVC (p = 0.054), while high FeNO was associated with normal lung function (p = 0.033) and less radiological severity (p = 0.012). Cardiovascular disease was more common in patients with high FeNO (p = 0.024), and allergic rhinitis was notably prevalent in those with increased BEC (p = 0.001). Treatment patterns varied, with patients having high FeNO less often on dual bronchodilator therapy (p = 0.017); however, no significant differences in ICS or prophylactic macrolide use were observed across the biomarker stratifications.

**Table 1:** Demographics for bronchiectasis patients stratified by baseline blood eosinophil counts, FeNO, and total IgE

Type 2 Biomarkers	BEC < 300 cells/μL	BEC ≥ 300 cells/μL	p value	FeNO < 25 ppb	FeNO ≥ 25 ppb	p value	IgE < 75 kU/L	IgE > 75 kU/L	p value
Number, (%)	110 (84.6)	20 (15.3)		96 (73.8)	34 (26.1)		82 (63.0)	48 (36.9)	
Age, years, median (IQR)	69.6 (61.8–76.8)	70.5 (58.4–79.5)	0.964	69.1 (59.7–77.7)	71.8 (66.2–75.9)	0.294	68.4 (59.9–75.0)	72.9 (62.8–78.6)	0.054
Gender, Male, n (%)	57 (51.8)	17 (85.0)	0.006*	50 (52.1)	24 (70.6)	0.061	40 (48.8)	34 (70.8)	0.014*
BMI, median (IQR)	20.6 (18.4–23.5)	25.2 (21.4–27.9)	< 0.001*	20.6 (19.2–23.4)	23.2 (19.9–27.3)	0.005*	20.5 (18.3–24.0)	21.8 (19.9–25.3)	0.094
Smoking status, n (%)									
Nonsmoker	72 (65.5)	7 (35.0)	0.010*	60 (62.5)	19 (55.9)	0.497	56 (68.3)	23 (47.9)	0.022*
Ex-or current smokers	38 (34.5)	13 (65.0)		36 (37.5)	15 (44.1)		26 (31.7)	25 (52.1)	
CAT score, median (IQR)	6 (3.0–12.0)	8.5 (3.2–12.7)	0.522	6.5 (3.0–12.0)	6.0 (1.7–11.2)	0.440	7.0 (3.0–12.0)	5.5 (2.0–10.0)	0.058
mMRC, median (IQR)	1 (1–2)	2 (1–2)	0.064	1 (1–2)	1 (1–2)	0.942	1 (0–2)	1 (1–2)	0.093
Exacerbation in the prior year, n (%)									
0–1 time/year	107 (97.3)	20 (100)	1.000	94 (97.9)	33 (97.1)	1.000	80 (97.6)	47 (97.9)	1.000
> 2 times	3 (2.7)	0 (0)		2 (2.1)	1 (2.9)		2 (2.4)	1 (2.1)	
Etiologies of bronchiectasis, n (%)									
Idiopathic	37 (33.6)	5 (25.0)	0.190	31 (32.3)	11 (32.4)	0.952	31 (37.8)	11 (22.9)	0.315
Post-infection									
Pneumonia	19 (17.3)	7 (35.0)		20 (20.8)	6 (17.6)		14 (17.1)	12 (25.0)	
NTM/TB	33 (30.0)	3 (15.0)		27 (28.1)	9 (26.5)		20 (24.4)	16 (33.3)	
COPD	14 (12.7)	5 (25.0)		13 (13.5)	6 (17.6)		11 (13.4)	8 (16.7)	
Autoimmune disease	5 (4.5)	0 (0)		4 (4.2)	1 (2.9)		4 (4.9)	1 (2.1)	
GERD	2 (1.8)	0 (0)		1 (1.0)	1 (2.9)		2 (2.4)	0 (0)	

For each row, data are either % with p-values from t test or Fisher’s exact tests between the two groups, median (IQR) with p-values from Mann‐Whitney tests

*BEC* blood eosinophil counts, *BMI* body mass index, *FeNO* fractional exhaled nitric oxide, *COPD* chronic obstructive pulmonary disease, *CAT* COPD assessment test, *GERD* gastroesophageal reflux disease, *IgE* immunoglobulin E, *mMRC* modified Medical Research Council, *TB* tuberculosis, *NTM* non-tuberculosis mycobacteria

*p < 0.05

**Table 2:** Lung function, radiological severity, and clinical profiles in bronchiectasis patients, categorized by baseline BEC, FeNO, and total IgE

Type 2 biomarkers	BEC < 300 cells/μL	BEC ≥ 300 cells/μL	p value	FeNO < 25 ppb	FeNO ≥ 25 ppb	p value	IgE < 75 kU/L	IgE ≥ 75 kU/L	p value
Number, (%)	110 (84.6)	20 (15.3)		96 (73.8)	34 (26.1)		82 (63.0)	48 (36.9)	
Lung function, median (IQR)									
FEV_1_/FVC (%)	71.6 (64.9–79.1)	66.1 (61.1–75.1)	0.054	69.9 (63.5–78.1)	75.4 (68.4–79.4)	0.089	73.5 (66.5–79.9)	66.5 (62.1–75.2)	0.003*
FEV_1_ (%)	80.6 (67.4–96.8)	67.3 (59.5–92.5)	0.160	77.0 (64.1–94.2)	87.1 (71.0–101.9)	0.084	80.6 (63.9–97.9)	77.6 (65.1–93.7)	0.806
FVC (%)	93.3 (80.0–101.8)	80.2 (75.5–100.9)	0.234	89.1 (76.0–100.6)	94.3 (84.8–102.7)	0.302	90.4 (74.3–101.3)	91.4 (79.6–105.7)	0.346
Normal, n (%)	40 (36.4)	5 (25.0)	0.611	27 (28.1)	18 (52.9)	0.033*	33 (40.2)	12 (25.0)	0.059
Obstructive pattern, n (%)	57 (51.8)	12 (60.0)		56 (58.3)	13 (38.2)		37 (45.1)	32 (66.7)	
Restrictive pattern, n (%)	13 (11.8)	3 (15.0)		13 (13.5)	3 (8.8)		12 (14.6)	4 (8.3)	
Image score									
Modified Reiff score, median (IQR)	4.0 (2.0–5.0)	4.5 (3.0–6.7)	0.095	4.0 (3.0–6.0)	3.0 (2.0–4.2)	0.012*	4.0 (2.0–5.0)	4.0 (3.0–5.7)	0.331
Cystic bronchiectasis, n (%)	12 (10.9)	5 (25.0)	0.086	14 (14.6)	3 (8.8)	0.557	10 (12.2)	7 (14.6)	0.697
BSI, median (IQR)	7.0 (5.0–10.0)	7.5 (4.0–10.7)	0.928	7.0 (5.0–11.0)	6.0 (4.0–9.0)	0.106	6.0 (4.0–9.0)	7.5 (5.0–12.5)	0.171
Mild (0–4), n (%)	26 (23.6)	6 (30.0)	0.677	21 (21.9)	11 (32.4)	0.277	22 (26.8)	10 (20.8)	0.630
Moderate (5–8), n (%)	44 (40.0)	6 (30.0)		36 (37.5)	14 (41.2)		32 (39.0)	18 (37.5)	
Severe (≥ 9), n (%)	40 (36.4)	8 (40.0)		39 (40.6)	9 (26.5)		28 (34.1)	20 (41.7)	
E-FACED, median (IQR)	2.0 (1.0–4.0)	3.0 (0.2–5.0)	0.579	3.0 (1.0–4.0)	2.0 (0.75–4.0)	0.385	2.0 (0.75–4.0)	3.0 (1.0–4.0)	0.077
Mild (0–3), n (%)	75 (68.2)	12 (60.0)	0.431	63 (65.6)	24 (70.6)	0.469	58 (70.7)	29 (60.4)	0.468
Moderate (4–6), n (%)	31 (28.2)	8 (40.0)		29 (30.2)	10 (29.4)		22 (26.8)	17 (35.4)	
Severe (7–9), n (%)	4 (3.6)	0 (0)		4 (4.2)	0 (0)		2 (2.4)	2 (42)	
Comorbidities, n (%)									
Cardiovascular disease	39 (35.5)	9 (45.0)	0.416	30 (31.3)	18 (52.9)	0.024*	29 (35.4)	19 (39.6)	0.631
Diabetes Mellitus	9 (11.4)	5 (9.8)	0.229	10 (10.4)	4 (11.8)	0.759	7 (8.5)	7 (14.6)	0.283
Hyperlipidemia	13 (11.8)	1 (5.0)	0.694	10 (10.4)	4 (11.8)	0.759	8 (9.8)	6 (12.5)	0.626
Old tuberculosis infection	33 (30.0)	3 (15.0)	0.276	27 (28.1)	9 (26.5)	0.853	20 (24.4)	16 (33.3)	0.271
Autoimmune disease	10 (9.1)	1 (5.0)	1.000	9 (9.4)	2 (5.9)	0.727	8 (9.8)	3 (6.3)	0.745
Reflux disease	53 (48.2)	5 (25.0)	0.055	37 (38.5)	21 (61.8)	0.019*	40 (48.8)	18 (37.5)	0.212
Allergic rhinitis	38 (34.5)	15 (75.0)	0.001*	37 (38.5)	16 (47.1)	0.385	33 (40.2)	20 (41.7)	0.873
Inhalation therapy, n (%) at baseline									
Monotherapy (LAMA or LABA)	17 (15.5)	3 (15.0)	1.000	15 (15.6)	5 (14.7)	0.898	13 (15.9)	7 (14.6)	0.846
LAMA + LABA	41 (37.3)	8 (40.0)	0.817	42 (43.8)	7 (20.6)	0.017*	27 (32.9)	22 (45.8)	0.143
Triple therapy	6 (5.5)	1 (5.0)	1.000	6 (6.3)	1 (2.9)	0.675	4 (4.9)	3 (6.3)	0.709
Inhaled corticosteroid (ICS)	8 (7.3)	4 (20.0)	0.089	10 (10.4)	2 (5.9)	0.731	5 (6.1)	7 (14.6)	0.107
Previous macrolides exposure, n (%)	13 (11.8)	1 (5.0)	0.694	10 (10.4)	4 (11.8)	0.759	9 (11.0)	5 (10.4)	0.921

Data are presented as No. (%) or median (interquartile range, IQR), unless otherwise indicated; For each row, data are either % with p-values from t test or Fisher’s exact tests between the two groups, median (IQR) with p-values from Mann‐Whitney tests

*BEC* blood eosinophil counts, *BSI* bronchiectasis severity index, *E-FACED* exacerbation, forced expiratory volume in 1 s (FEV_1_), age, chronic colonization by *Pseudomonas aeruginos*a, radiological extension and dyspnea, *FeNO* fractional exhaled nitric oxide, *FEV*_*1*_ forced expiratory volume in 1 s, *FVC* forced vital capacity, *IgE* immunoglobulin E, *LAMA* long-acting muscarinic antagonist, *LABA* long-acting β2 sympathomimetic agonists, *ICS* inhaled corticosteroid

*p < 0.05

### Laboratory, Microbiological, and Clinical Outcomes by Type 2 Biomarkers in Bronchiectasis

Table 3 delineates the laboratory and microbiological findings in bronchiectasis patients, categorized by Type 2 biomarker status. Patients with BEC ≥ 300 cells/μL exhibited elevated IgE levels (p < 0.001, p = 0.035). Elevated BEC were also observed in those with IgE ≥ 75 kU/L (p < 0.001, p = 0.004). In contrast, FeNO ≥ 25 ppb correlated with lower neutrophil counts (p = 0.035), with no significant correlation to BEC or IgE, aligning with the findings depicted in Fig. 1B–D. Furthermore, a higher percentage of BAL lymphocytes was observed in patients with FeNO ≥ 25 ppb (p = 0.015), alongside significantly diminished cytokine concentrations (IL-1β, IL-6, IL-8, TNF-α). However, no notable differences were found in BAL macrophage, neutrophil, or eosinophil counts across biomarker statuses. Correlational analysis of BAL eosinophils with FeNO, BEC, and serum IgE revealed weak and statistically insignificant relationships. FeNO was marginally inversely related to BAL eosinophils (r = − 0.138), while BEC and IgE had slight positive correlations (r = 0.113 and r = 0.094, respectively), all lacking statistical significance (p > 0.05) as illustrated in Fig. S1. Predominant pathogens included *Klebsiella pneumoniae* (33.0%), *Pseudomonas aeruginosa* (26.9%), and others, with their prevalence consistent irrespective of biomarker levels. Clinically, the BEC ≥ 300 cells/μL group showed a trend towards more frequent exacerbations, nearing significance (p = 0.050), whereas patients with FeNO ≥ 25 ppb experienced significantly fewer exacerbations (p = 0.048).

**Table 3:** Comparison of laboratory results and outcomes based on baseline BEC, FeNO, and total IgE (N = 130)

Biomarkers	BEC < 300 cells/μL	BEC ≥ 300 cells/μL	p value	FeNO < 25 ppb	FeNO ≥ 25 ppb	p value	IgE < 75 kU/L	IgE ≥ 75 kU/L	p value
Number, (%)	110(84.6)	20 (15.3)		96 (73.8)	34 (26.1)		82 (63.0)	48 (36.9)	
Blood samples, median (IQR)									
Neutrophils counts (cells/mm^3^)	3882 (2937–5127)	4730 (3321–5891)	0.061	4349 (3064–5668)	3474 (2703–4336)	0.035*	3975 (3014–4986)	4225 (3088–5963)	0.265
Eosinophil counts (cells/mm^3^)	143 (72–202)	414 (335–475)	< 0.001*	150 (80–246)	135 (74–229)	0.628	130 (60–227)	177 (131–308)	0.004*
Lymphocyte counts(cells/mm^3^)	1772 (1334–2193)	1692 (1365–1898)	0.638	1755 (1365–2094)	1721 (1310–2336)	0.84	1750 (1426–2193)	1744 (1264–2106)	0.689
Neutrophil–lymphocyte ratio	2.22 (1.56–3.24)	2.69 (1.80–4.24)	0.083	2.57 (1.66–3.65)	1.89 (1.36–3.00)	0.057	2.23 (1.52–3.24)	2.52 (1.76–3.57)	0.224
C-reactive protein (mg/dL)	0.19 (0.06–0.75)	0.30 (0.25–0.77)	0.154	0.32 (0.09–9.86)	0.14 (0.07–0.32)	0.053	0.18 (0.07–0.72)	0.31 (0.09–0.97)	0.163
IgE (kU/L)	47.3 (18.4–98.9)	87.2 (35.2–225.5)	0.035*	56.5 (22.7–129.2)	36.8 (12.2–76.3)	0.108	25.1 (10.5–48.3)	154.0 (94.5–287.5)	< 0.001*
FeNO, ppb	17.0 (11.7–25.2)	19.5 (14.5–24.0)	0.429	15.0 (10.0–19.0)	30.0 (27.7–40.0)	< 0.001*	18.5 (12.0–26.2)	16.0 (13.0–23.7)	0.395
BAL samples, median (IQR)									
BAL macrophage (%)	86.5 (78.3–90.5)	87.9 (82.1–91.5)	0.296	87.2 (80.9–91.5)	86.0 (73.9–89.9)	0.114	86.8 (79.0–91.2)	86.7 (78.4–90.4)	0.981
BAL neutrophil (%)	1.61 (0.79–4.34)	2.29 (0.97–4.60)	0.273	1.83 (0.83–4.48)	1.91 (0.88–4.30)	0.924	1.98 (0.88–4.19)	1.37 (0.82–4.91)	0.446
BAL eosinophil (%)	2.2 (1.1–4.0)	2.4 (1.5–5.0)	0.458	2.1 (1.1–3.9)	2.4 (1.5–5.0)	0.309	2.22 (1.21–3.77)	2.23 (1.32–4.52)	0.725
BAL lymphocyte (%)	7.8 (3.4–14.1)	5.8 (2.6–8.0)	0.079	6.5 (3.0–10.6)	9.4 (5.8–17.3)	0.015*	7.09 (2.95–13.4)	7.97 (3.95–11.53)	0.858
BAL IL-1β (pg/mL)	24.2 (4.0–266.6)	26.6 (4.5–196.8)	0.897	38.2 (5.3–292.4)	7.05 (2.2–47.0)	0.005*	27.7 (4.5–239.5)	18.8 (3.8–248.7)	0.647
BAL IL-6 (pg/mL)	27.6 (5.9–54.7)	18.4 (4.1–77.1)	0.603	31.7 (6.9–68.4)	8.3 (1.2–36.4)	0.005*	23.5 (6.0–56.0)	25.1 (5.4–48.5)	0.836
BAL IL-8 (pg/mL)	563.6 (189.7–1689.7)	962.0 (140.5–2066.9)	0.816	861.8 (258.0–1837.1)	322.9 (46.6–1522.0)	0.017*	767.7 (211.2–1772.5)	440.4 (159.4–1657.2)	0.616
BAL TNF-α (pg/mL)	10.8 (4.3–33.6)	8.6 (6.3–25.7)	0.895	11.4 (5.2–38.6)	6.1 (1.2–20.5)	0.016*	11.1 (4.2–33.6)	8.1 (4.9–27.6)	0.669
BAL MCP-1 (pg/mL)	255.1 (166.9–567.0)	203.2 (85.6–524.3)	0.175	259.6 (163.9–645.5)	228.2 (117.4–402.6)	0.090	237.8 (140.2–564.6)	272.3 (165.6–567.1)	0.664
BAL conventional culture									
*Klebsiella pneumoniae,* n (%)	37 (33.6)	6 (30.0)	0.751	31 (32.3)	12 (35.3)	0.749	27 (32.9)	16 (33.3)	0.962
*Pseudomonas aeroginosa,* n (%)	31 (28.2)	4 (20.0)	0.588	27 (28.1)	8 (23.5)	0.604	21 (25.6)	14 (29.2)	0.659
*Staphylococcus aureus,* n (%)	19 (17.3)	2 (10.0)	0.527	16 (16.7)	5 (14.7)	0.789	14 (17.1)	7 (14.6)	0.710
*Haemophilus influenzae,* n (%)	8 (7.3)	3 (15.0)	0.374	6 (6.3)	5 (14.7)	0.128	6 (7.3)	5 (10.4)	0.540
NTM n (%)	20 (18.2)	1 (5.0)	0.195	16 (16.7)	5 (14.7)	0.789	16 (19.5)	5 (10.4)	0.174
*Aspergillus species,* n (%)	9 (8.2)	2 (10.0)	0.677	10 (10.4)	1 (2.9)	0.286	6 (7.3)	5 (10.4)	0.540
*Candida species,* n (%)	31 (28.2)	5 (25.0)	0.770	27 (28.1)	9 (26.5)	0.853	18 (22.0)	18 (37.5)	0.056
Clinical outcomes									
Severe exacerbation, n (%)	29 (26.4)	8 (40.0)	0.214	31 (32.3)	6 (17.6)	0.104	26 (31.7)	11 (22.9)	0.284
Moderate/severe exacerbation, n (%)	41 (37.3)	12 (60.0)	0.050	44 (45.8)	9 (26.5)	0.048*	34 (41.5)	19 (39.6)	0.833

Data are presented as No. (%) or median (interquartile range, IQR), unless otherwise indicated; For each row, data are either % with p-values from t test or Fisher’s exact tests between the two groups, median (IQR) with p-values from Mann‐Whitney tests

*BAL* bronchoalveolar lavage, *BEC* blood eosinophil counts, *FeNO* fractional exhaled nitric oxide, *IgE* immunoglobulin E, *NLR* neutrophil–lymphocyte ratio, *NTM* non-tuberculosis mycobacteria, *IL-1β* interleukin [IL]-1β, *IL-6* interleukin [IL]-6, *IL-8* interleukin [IL]-8, *MCP-1* monocyte chemoattractant protein-1, *TNF-α* tumor necrosis factor [TNF]-α

*p < 0.05

#### Correlations of Type 2 Biomarkers with Inflammatory Markers and Disease Severity in Bronchiectasis

**Fig. 2:**
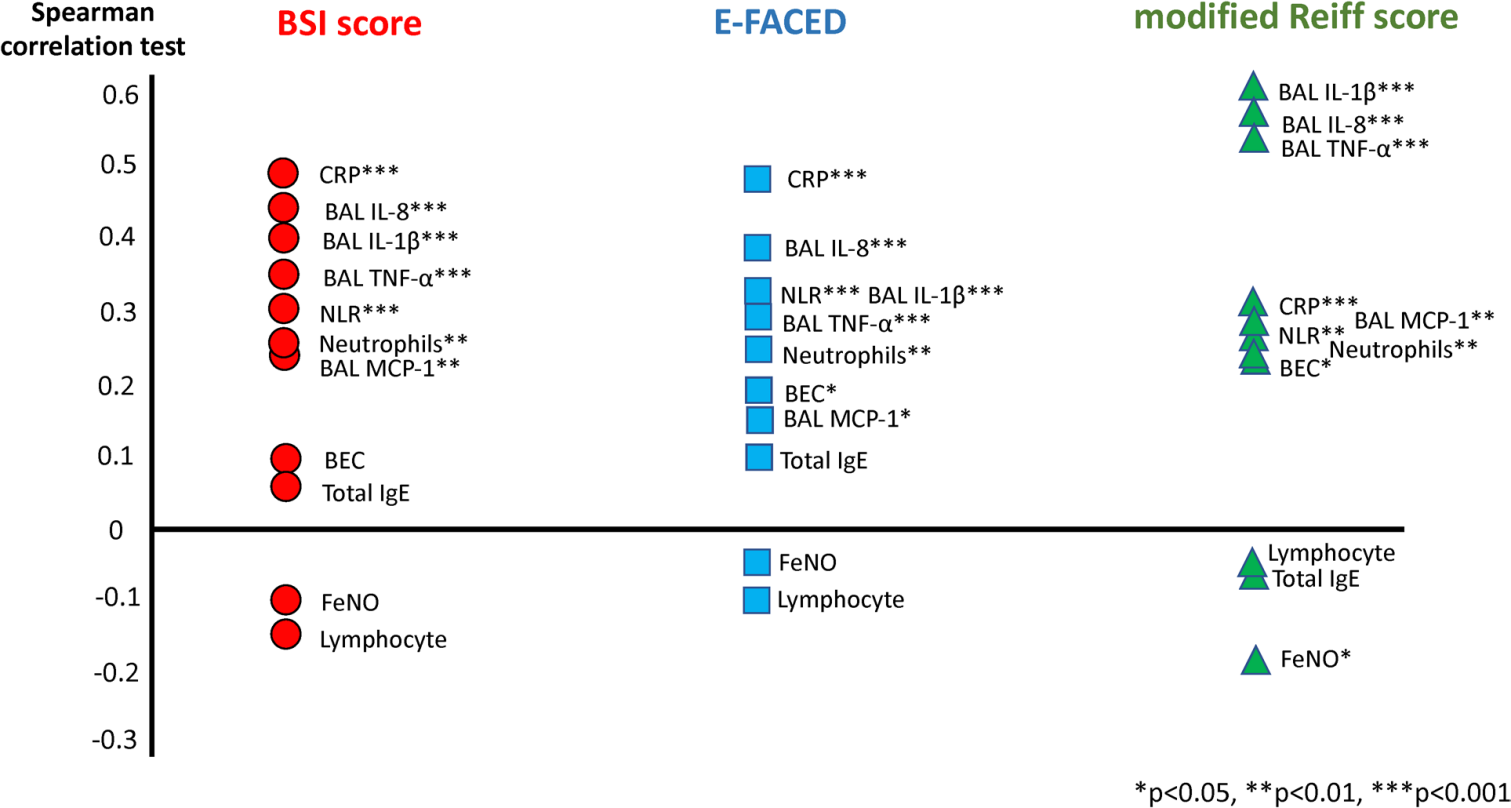
The correlations between Type 2 biomarkers, inflammatory markers, and immunological profiles with the severity scores in bronchiectasis. Spearman’s test assessed correlations between quantitative variables. The presence of asterisks indicates the level of statistical significance, with (*) denoting p < 0.05, (**) indicating p < 0.01, and (***) representing p < 0.001. The direction of the correlations is represented by the position of the markers: above the zero line for positive correlations and below for negative correlations. *BAL* bronchoalveolar lavage, *BEC* blood eosinophil counts, *BSI* bronchiectasis severity index, *CRP* C reactive protein, *E-FACED* exacerbation, forced expiratory volume in 1 s (FEV_1_), age, chronic colonization by *Pseudomonas aeruginos*a, radiological extension and dyspnea, *FeNO* fractional exhaled nitric oxide, *IgE* immunoglobulin E, *IL-1β* interleukin [IL]-1beta, *IL-6* interleukin[IL]-6, *IL-8* interleukin [IL]-8, *MCP-1* monocyte chemoattractant protein-1, *NLR* neutrophil–lymphocyte ratio, *TNF-α* tumor necrosis factor-alpha

Figure 2 illustrates the relationships between Type 2 biomarkers, inflammatory markers, immunological profiles, and bronchiectasis severity. BEC showed a positive correlation with severity, notably with E-FACED (r = 0.190, p = 0.03) and modified Reiff scores (r = 0.222, p = 0.011). In contrast, FeNO was negatively correlated with severity, significant only for the modified Reiff score (r = − 0.181, p = 0.04). Serum total IgE levels did not significantly correlate with severity scores. Additionally, local (BAL IL-1β, IL-6, IL-8, TNF-α) and systemic markers [C-reactive protein (CRP), neutrophil-to-lymphocyte ratio (NLR), and neutrophils] demonstrated stronger positive correlations with severity than Type 2 biomarkers (Table S1).

#### Exacerbation Risk Prediction in Bronchiectasis Using Composite Type 2 Biomarkers

**Fig. 3:**
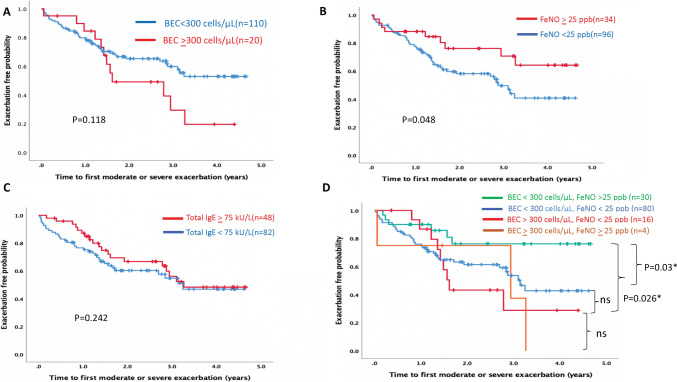
Kaplan–Meier curves illustrate the risk of moderate or severe exacerbations in bronchiectasis patients, stratified by: **A** BEC levels ≥ 300 cells/μL or < 300 cells/μL, **B** FeNO levels ≥ 25 ppb or < 25 ppb, **C** serum total IgE levels ≥ 75 kU/L or < 75 kU/L, and **D** combined BEC and FeNO into four subgroup levels. *BEC* blood eosinophil counts, *IgE* immunoglobulin E, *FeNO* fractional exhaled nitric oxide

Exploring the differential impacts of FeNO and BEC on exacerbation risk, we analyzed clinical variables alongside bronchiectasis severity and exacerbation rates, stratified by BEC and FeNO thresholds (Table 4). Patients with BEC ≥ 300 cells/μL and FeNO < 25 ppb showed elevated modified Reiff scores compared to those with opposite biomarker levels (p = 0.025), while BSI and E-FACED scores remained consistent. BAL markers IL-1β, IL-6, and IL-8, linked to severity in Fig. 2, were elevated in patients with high BEC (≥ 300 cells/μL) and low FeNO (< 25 ppb) (Table 4). Longitudinal analysis with a median follow-up time of 2.76 years for exacerbation outcomes indicated that high FeNO (≥ 25 ppb) was associated with reduced exacerbation risks (p = 0.048) (Fig. 3B), whereas high BEC (≥ 300 cells/μL) tended toward an increased risk, albeit not significantly (p = 0.118) (Fig. 3A). Serum total IgE levels did not significantly correlate with exacerbation risk (Fig. 3C). Subgroup analysis highlighted that low BEC and high FeNO were predictive of fewer exacerbations, especially when compared to high BEC/low FeNO (p = 0.026) and low BEC/low FeNO groups (p = 0.03) (Fig. 3D).

**Table 4:** Clinical variables, severity of bronchiectasis, and exacerbations for bronchiectasis patients in groups stratified by blood eosinophil counts and FeNO

Biomarkers	BEC < 300 cells/μL, FeNO < 25 ppb	BEC < 300 cells/μL, FeNO ≥ 25 ppb	BEC ≥ 300 cells/μL, FeNO < 25 ppb	BEC ≥ 300 cells/μL, FeNO ≥ 25 ppb	p value
Number	80	30	16	4	
Lung function					
Normal, n (%)	23 (28.7)	17 (56.7)	4 (25.0)	1 (25.0)	0.170
Obstructive pattern, n (%)	46 (57.5)	11 (36.7)	10 (62.5)	2 (50.0)	
Restrictive pattern, n (%)	11 (13.8)	2 (6.7)	2 (12.5)	1 (25.0)	
Image score					
Modified Reiff score (0–18), median (IQR)	4.0 (2.0–5.0)	3.0 (2.0–4.25)	5.0 (3.0–8.5)	2.5 (2.0–5.2)	0.025
Cystic bronchiectasis, n (%)	10 (12.5)	2 (6.7)	4 (25.0)	1 (25.0)	0.307
Bronchiectasis severity index, median (IQR)	7.0 (5.0–10.7)	6.0 (4.0–9.0)	8.0 (5.0–11.75)	4.0 (1.7–8.5)	0.302
E-FACED, median (IQR)	2.0 (1.0–4.0)	2.0 (1.0–4.0)	3.0 (1.0–5.0)	1.0 (0–5.0)	0.632
Blood samples, median (IQR)					
Neutrophil–lymphocyte ratio (NLR)	2.4 (1.6–3.3)	1.8 (1.2–2.9)	2.6 (1.8–4.3)	2.7 (1.8–3.3)	0.095
BAL samples, median (IQR)					
BAL IL-1β (pg/mL)	36.6 (5.3–292.4)	11.2 (2.4–103.9)	59.6 (5.9–314.7)	3.0 (0.6–7.0)	0.017
BAL IL-6 (pg/mL)	32.7 (6.9–66.4)	9.7 (2.6–37.9)	25.6 (6.0–93.8)	2.6 (0–24.4)	0.026
BAL IL-8 (pg/mL)	767.7 (247.1–1804.1)	337.4 (97.7–1568.2)	1290.1 (264.3–2279.2)	39.0 (10.5–533.0)	0.023
BAL TNF-α (pg/mL)	12.6 (4.8–43.9)	6.1 (1.9–21.1)	8.6 (6.9–31.0)	5.6 (0–11.5)	0.092
BAL MCP-1 (pg/mL)	265.3 (170.2–778.8)	233.2 (158.8–402.6)	224.6 (123.1–567.1)	94.6 (23.6–380.4)	0.139
Baseline medications, n (%)					
Dual bronchodilators	34 (42.5)	7 (23.3)	8 (50.0)	0 (0)	0.076
Triple therapy	6 (7.5)	0 (0)	0 (0)	1 (25)	0.096
Inhaled corticosteroid (ICS)	8 (10.0)	0 (0)	2 (12.5)	2 (50.0)	0.010
Previous macrolides exposure	9 (11.3)	4 (13.3)	1 (6.3)	0 (0)	0.790
Clinical outcomes					
Severe exacerbation, n (%)	26 (32.5)	3 (10.0)	5 (31.3)	3 (75.0)	0.019
Moderate or severe exacerbation, n (%)	35 (34.8)	6 (20.0)	9 (56.3)	3 (75.0)	0.027

Data are presented as No. (%) or median (interquartile range, IQR), unless otherwise indicated. Kruskal–Wallis was applied between multiple groups of data, and categorical variables with the chi-squared or Fisher’s exact tests

*BAL* bronchoalveolar lavage, *BEC* blood eosinophil counts, *BSI* bronchiectasis severity index,* E-FACED* exacerbation, forced expiratory volume in 1 s (FEV_1_), age, chronic colonization by *Pseudomonas aeruginos*a, radiological extension and dyspnea, *FeNO* fractional exhaled nitric oxide, *ICS* inhaled corticosteroid, *IL-1β* interleukin [IL]-1beta, *IL-6* interleukin[IL]-6, *IL-8* interleukin [IL]-8, *MCP-1* monocyte chemoattractant protein-1, *NLR* neutrophil–lymphocyte ratio, *NTM* non-tubercuosis mycobacteria, *TNF-α* tumor necrosis factor-alpha

#### Type 2 Biomarkers and Clinical Indicators as Exacerbation Predictors in Bronchiectasis

**Fig. 4:**
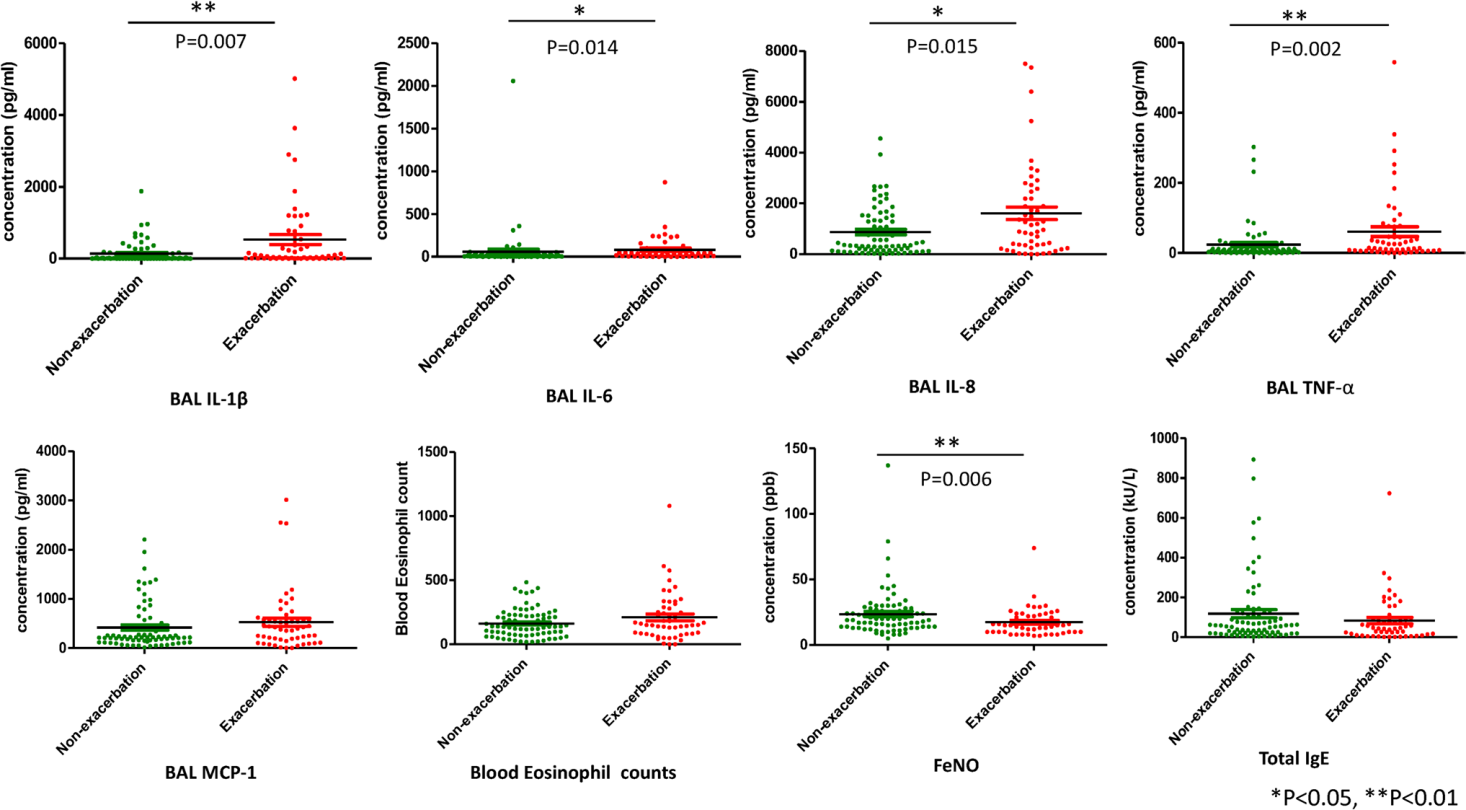
Type 2 biomarkers and airway cytokines in exacerbation and non-exacerbation groups of bronchiectasis patients. *IL-6* interleukin-6, *IL-8* interleukin-8, *IL-1β* interleukin-1 beta, *TNF-α* tumor necrosis factor-alpha, *BEC* blood eosinophil count, *IgE* immunoglobulin E, *FeNO* fractional exhaled nitric oxide

**Fig. 5:**
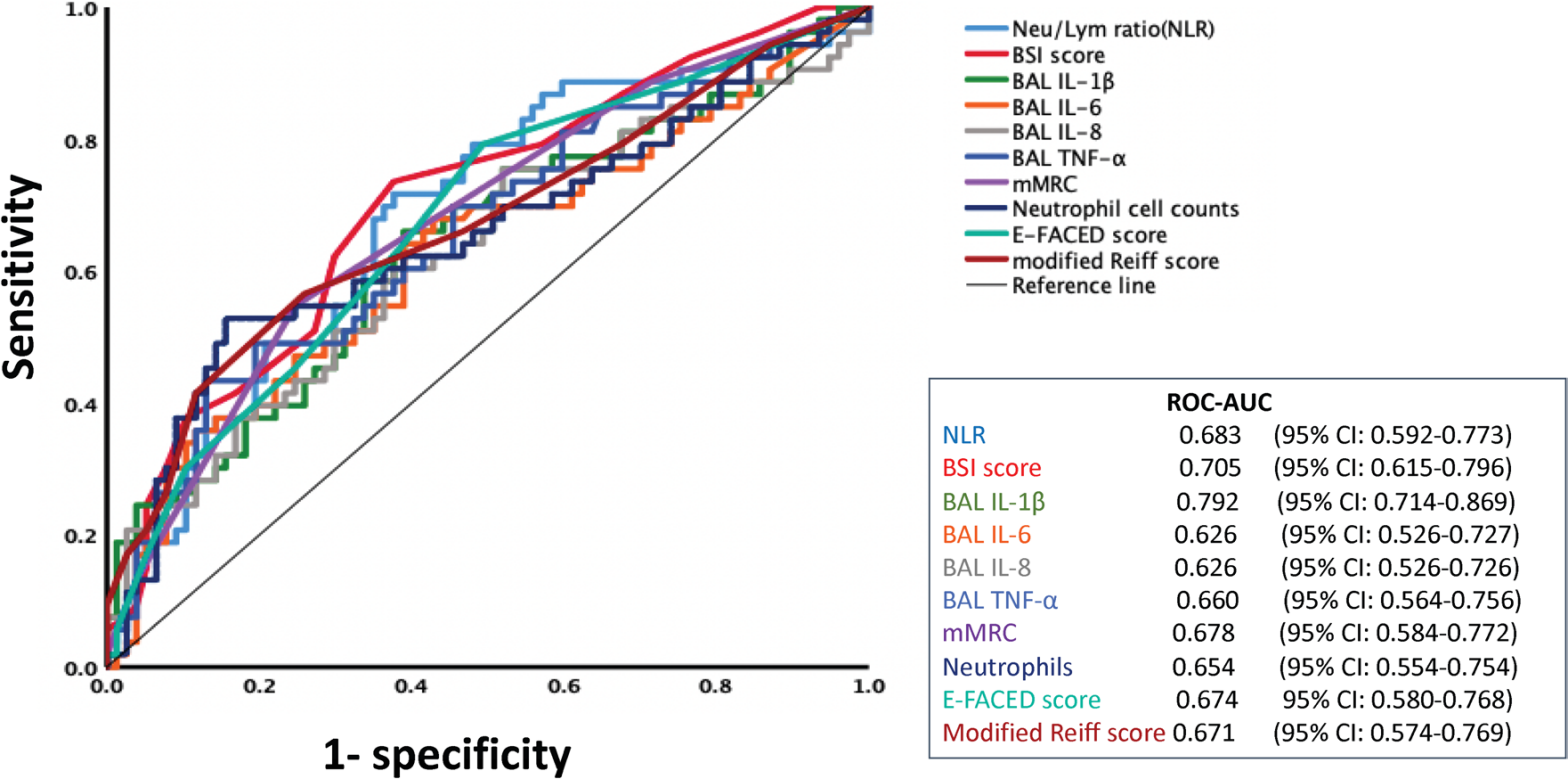
Receiver operating characteristic (ROC) curve and area under the curve (AUC) with 95% confidence intervals (CIs) for clinical variables and biomarkers predictive of future exacerbations. *BAL* bronchoalveolar lavage, *BSI* bronchiectasis severity index, *IL-6* interleukin[IL]-6, *IL-8* interleukin [IL]-8, *IL-1β* interleukin [IL]-1beta, *mMRC* modified Medical Research Council, *NLR* neutrophil–lymphocyte ratio, *TNF-α* tumor necrosis factor-alpha,* E-FACED* exacerbation, forced expiratory volume in 1 s (FEV_1_), age, chronic colonization by *Pseudomonas aeruginos*a, radiological extension and dyspnea

Figure 4 stratifies bronchiectasis patients by exacerbation status, showing that those with higher FeNO levels experience fewer exacerbations (p = 0.006) and those with exacerbations present with elevated BAL cytokines (IL-6, IL-8, IL-1β, TNF-α). ROC analysis indicates BAL IL-1β as the strongest predictor of exacerbations (AUC: 0.792), alongside significant BSI scores and NLR (Fig. 5). Figs. S2 and S3 highlight clinical factors that increase exacerbation risk, including *Pseudomonas aeruginosa* colonization, FEV1/FVC < 0.7, high NLR [NLR ≥ 3.0, cutoff value based on the mean NLR (2.9 ± 2.2, p < 0.001)], elevated BSI scores (≥ 9), mMRC dyspnea scale (≥ 2), prior macrolide therapy, and current inhalation therapies, but not ICS use. Multivariable analysis in Table 5 designates *Pseudomonas aeruginosa* colonization and high NLR (≥ 3.0) as independent predictors of exacerbations with HRs of 2.065 (p = 0.035) and 1.877 (p = 0.048), respectively. The combined low BEC and high FeNO indicated a decreased risk in univariable analysis but did not reach statistical significance in the multivariable analysis (HR: 0.459, p = 0.098).

**Table 5:** Multivariable analysis of key clinical factors associated with moderate to severe exacerbations in patients with bronchiectasis

Clinical factors	Univariable analysis		Multivariable analysis	
	HR (95% CI)	p value	HR (95% CI)	p value
mMRC ≥ 2	2.378 (1.383–4.089)	0.002*	1.328 (0.660–2.668)	0.426
FEV_1_/FVC < 0.7	1.877 (1.082–3.256)	0.025*	1.230 (0.651–2.326)	0.524
Modified Reiff score	1.201 (1.102–1.309)	< 0.001*	1.074 (0.956–1.205)	0.228
Neutrophil–lymphocyte ratio (NLR ≥ 3.0)	2.900 (1.679–5.011)	< 0.001*	1.877 (1.004–3.508)	0.048*
BAL IL-1β (pg/mL)	1.000 (1.000–1.001)	< 0.001*	1.000 (1.000–1.001)	0.283
BAL TNF-α (pg/mL)	1.003 (1.001–1.006)	0.002*	0.999 (0.993–1.006)	0.831
Composite Type 2 biomarkers				
BEC < 300 cells/μL and FeNO < 25 ppb	1.000		1.000	
BEC < 300 cells/μL and FeNO ≥ 25 ppb	0.386 (0.162–0.920)	0.032*	0.459 (0.182–1.154)	0.098
BEC ≥ 300 cells/μL and FeNO < 25 ppb	1.297 (0.622–2.703)	0.488	0.881 (0.374–2.071)	0.771
BEC ≥ 300 cells/μL and FeNO ≥ 25 ppb	1.554 (0.477–5.067)	0.464	2.939 (0.574–15.052)	0.196
*Pseudomonas aeroginosa*	1.903 (1.091–3.319)	0.023*	2.065 (1.051–4.057)	0.035*
Inhalation therapy	2.182 (1.165–4.089)	0.015*	1.315 (0.589–2.937)	0.505
Previous macrolide use	2.462 (1.091–5.557)	0.03*	1.407 (0.515–3.847)	0.505
Bronchiectasis severity index score				
Mild (0–4)	1.000		1.000	
Moderate (5–8)	2.014 (0.846–4.796)	0.114	1.839 (0.583–5.804)	0.299
Severe (≥ 9)	2.997 (1.304–6.885)	0.010*	1.345 (0.367–4.929)	0.654

*BAL* bronchoalveolar lavage, *BEC* blood eosinophil counts, *BMI* body mass index, *CI* confidence interval, *FeNO* fractional exhaled nitric oxide, *FEV*_*1*_ forced expiratory volume in 1 s, *FVC* forced vital capacity, *HR* hazard ratio, *IL-1β* interleukin [IL]-1beta, *mMRC* modified Medical Research Council, *NLR* neutrophil–lymphocyte ratio, *TNF-α* tumor necrosis factor-alpha. Inhalation therapy includes monotherapy, dual therapy and triple therapy

*p < 0.05

## Discussion

Our study is the first to apply three Type 2 biomarkers (BEC, total IgE, and FeNO) in an East Asian bronchiectasis cohort, revealing that 60% of patients had at least one positive marker. The impacts of these biomarkers on disease severity and outcomes varied, indicating that BEC and FeNO may represent distinct aspects of disease severity. Notably, a combination of low BEC with high FeNO suggested lower disease severity and a trend towards lower exacerbation risk. However, *Pseudomonas aeruginosa* colonization and high NLR (≥ 3.0) were identified as stronger exacerbation predictors. These findings imply that the clinical and pathophysiological complexity of bronchiectasis may not be sufficiently explained by the binary classification of ‘Type 2 high’ or ‘Type 2 low’ that is based solely on traditional type 2 biomarkers, as commonly applied in asthma management. This study underscores the benefits of employing multi-biomarker strategies instead of relying on single markers for a more effective risk stratification and precise treatment of bronchiectasis.

Our study on Type 2 biomarkers within an East Asian bronchiectasis cohort reveals distinct inflammation patterns when compared to asthma [10] and COPD [27]. Defining Type 2 inflammation by the presence of any positive marker, we found that 60% of our patients exhibited such inflammation, a prevalence situated between asthma (88%) [10] and COPD (42.5%) [27]. Specifically, eosinophilic bronchiectasis (BEC ≥ 300 cells/µL) was present in 15.3% of patients, lower than the 22% seen in European cohorts [6], hinting at potential regional differences due to genetic or environmental factors [6, 28, 29]. Elevated FeNO levels (≥ 25 ppb) occurred in 26.1% of patients, less than the prevalence in COPD (42.8%) [30] and asthma (51%) [10], with weakened correlation with BEC possibly resulting from the exclusion of patients with asthma [31], significant bacterial colonization [32], or the inherent heterogeneity of bronchiectasis [33]. Additionally, 36.9% had high serum IgE levels (≥ 75 kU/L), slightly below the 43.2% identified with > 60 kU/L in other studies [8]. Contrary to the findings of Ren et al. [8], our data indicate a positive correlation between IgE and BEC rather than FeNO, and a non-significant negative correlation with FeNO, highlighting the complex interplay of Type 2 biomarkers in bronchiectasis. Moreover, our analysis showed only weak and non-significant correlations between BAL eosinophils and FeNO, BEC, and serum IgE (p > 0.05), suggesting that BAL eosinophil percentages do not correlate strongly with systemic biomarkers commonly associated with inflammation and allergic responses in bronchiectasis patients. Further research is warranted to elucidate these findings.

In our study, male bronchiectasis patients who smoked with fixed airflow obstruction demonstrated elevated BEC and IgE levels, paralleling observations in COPD [34–36] and bronchiectasis [8, 37]. This pattern suggests a connection between these biomarkers and reduced pulmonary function. Yet, radiological severity of bronchiectasis was associated only with BEC, not total IgE, diverging from prior findings [8, 37], despite the positive BEC-IgE correlation. In contrast, higher FeNO levels (≥ 25 ppb) correlated with less radiological severity and fewer obstructive patterns, a divergence from earlier reports [7, 8, 32], but consistent with a recent Chinese bronchiectasis cohort [38], illustrating variable FeNO impacts. Furthermore, patients with high FeNO had lower blood neutrophil counts and reduced levels of airway pro-inflammatory cytokines (IL-1β, IL-6, IL-8, and TNF-α), which may explain why elevated FeNO is associated with less severe disease and a lower risk of exacerbation. Further research is warranted to clarify the relationship between elevated FeNO and bronchiectasis severity, particularly in patients without concurrent asthma.

The findings elucidate the nuanced relationship between type 2 biomarkers and exacerbation risk in bronchiectasis. Elevated BEC (≥ 300 cells/µL) correlates with higher E-FACED scores and a tendency for more frequent exacerbations, whereas FeNO levels ≥ 25 ppb are linked to a lower risk. Notably, a combination of low BEC and high FeNO significantly reduced modified Reiff scores and exacerbation rates, suggesting that BEC and FeNO reflect distinct aspects of disease severity, as seen in a retrospective Chinese cohort [38], akin to findings in a prior COPD study [30]. Building on these observations, we infer that in eosinophilic COPD, particularly in cases of severe COPD and emphysema, reduced FeNO levels often result from severe peripheral airway narrowing or obliteration, or the development of emphysema [30, 39, 40]. Similar phenomena may be observed in bronchiectasis, where severe ciliary damage and excess mucus can sequester liberated FeNO in the lung peripheries, rendering it inaccessible for proximal airway measurement [41, 42]. This pattern suggests a shared pathophysiological mechanism in these conditions, where structural changes in the lungs can significantly impact FeNO levels.

However, the relationship between exacerbation risk and other biomarkers such as serum IgE levels is less straightforward. Notably, serum IgE levels did not show a strong correlation with exacerbation risk, challenging the predictability based on Type 2 biomarkers alone. Our research highlights BEC and FeNO as composite biomarkers for enhancing risk stratification in eosinophilic bronchiectasis, a treatable trait that predisposes patients to exacerbation risks [43, 44]. The potential benefits of therapies such as inhaled corticosteroids (ICS) [44–46] and anti-IL-5/anti-IL-5 receptor biologics [47] in mitigating exacerbations emphasize their importance for personalized treatment, pending further evaluation.

Despite 86.9% of our cohort showing pathogen colonization detected by BAL samples, no significant differences in microbiome profiles, including *Klebsiella pneumoniae* and *Pseudomonas aeruginosa*, were observed across Type 2 biomarker levels, consistent with prior studies [7, 8]. However, contrasts with European cohorts [6, 17] might stem from our use of conventional culture methods instead of next-generation sequencing (NGS) analysis [6, 17] and different inflammatory cluster classifications [17]. Our findings align with existing literature that identifies *Pseudomonas aeruginosa* colonization [2, 3], airflow limitation [2, 48, 49], increased airway neutrophilic inflammation, NLR [2, 3, 38, 50], and high BSI scores [2, 25] as significant predictors of exacerbations. Multivariable analysis identified *Pseudomonas aeruginosa* colonization and high NLR (≥ 3.0) as independent predictors of exacerbations. The interplay of low BEC and high FeNO hinted at reduced exacerbation risks, albeit without statistical significance. Our data indicate that while traditional risk factors like *Pseudomonas* colonization and high NLR are confirmed predictors of exacerbations, Type 2 biomarker combinations could enhance patient stratification, supporting a multi-biomarker approach in developing personalized bronchiectasis treatment plans [6–8, 17, 33].

The strength of this study lies in its comprehensive integration of three Type 2 biomarkers, systemic and local inflammatory markers, and microbiological data within a well-defined bronchiectasis cohort, with exclusion criteria applied to patients with clinical asthma and ABPA. However, it encounters several limitations. Firstly, the lack of research on T2-high endotypes and the establishment of thresholds for type 2 biomarkers in bronchiectasis underscores the need for consensus on diagnostic benchmarks. Secondly, its cross-sectional design precludes the observation of longitudinal changes in Type 2 biomarkers. Thirdly, the relatively small sample size in specific subgroups, especially those with low BEC and high FeNO, as well as in the eosinophilic bronchiectasis subgroup, limits our ability to fully evaluate their impact on exacerbation risks. This limitation underscores the need for further studies to confirm these preliminary findings and to enhance our understanding of their clinical implications. Fourthly, the absence of control groups constrains comparative analyses. Fifthly, reliance on conventional culture methods rather than NGS restricts the scope of our microbiome analysis. Sixthly, sample collection during stable disease states necessitates follow-up studies to monitor biomarker changes during exacerbations. Lastly, conducting this study within a Taiwanese East Asian population highlights the necessity for further validation across diverse geographical and ethnic contexts.

In conclusion, our investigation offers groundbreaking perspectives on Type 2 biomarkers within an East Asian bronchiectasis cohort, demonstrating the differential roles of BEC, FeNO, and serum IgE in influencing exacerbation risk. These findings highlight the multifaceted nature of bronchiectasis, advocating for holistic patient assessments that combine biomarker insights with airway microbiology and clinical indices. Our study reinforces the utility of multi-biomarker strategies, as opposed to single-marker methods, for nuanced risk stratification and individualized bronchiectasis treatment.

## Supplementary Information

Below is the link to the electronic supplementary material.

Supplementary file1 (DOCX 8103 KB)

## Data Availability

No datasets were generated or analysed during the current study.

## Abbreviations

AUC: Area under the curve
BAL: Bronchoalveolar lavage
BEC: Blood eosinophil counts
BMI: Body mass index
BSI: Bronchiectasis severity index
CAT: COPD assessment test
CIs: Confidence intervals
COPD: Chronic obstructive pulmonary disease
CRP: C reactive protein
E-FACED: Exacerbation, Forced expiratory volume in 1 s (FEV_1_), Age, Chronic colonization by *Pseudomonas aeruginos*a, Radiological extension and Dyspnea
FeNO: Fractional exhaled nitric oxide
FEV1: Forced expiratory volume in 1 s
FVC: Forced vital capacity
ICS: Inhaled corticosteroid
IL-1β: Interleukin [IL]-1β
IL-6: Interleukin [IL]-6
IL-8: Interleukin [IL]-8
IgE: Immunoglobulin E
LABA: Long-acting β2 sympathomimetic agonists
LAMA: Long-acting muscarinic antagonist
MCP-1: Monocyte chemoattractant protein-1
mMRC: Modified Medical Research Council
NLR: Neutrophil–lymphocyte ratio
NTM: Non-tuberculosis mycobacteria
ROC: Receiver operating characteristic
TB: Tuberculosis
TNF-α: Tumor necrosis factor [TNF]-α
