# Supplementary material for: Type 2 Biomarkers and Their Clinical Implications in Bronchiectasis: A Prospective Cohort Study
Source: Lung. 2024 Jun 17;202(5):695–709. doi: 10.1007/s00408-024-00707-0 (PMC11427600; doi:10.1007/s00408-024-00707-0)

**Type 2 Biomarkers and Their Clinical Implications in Bronchiectasis: A Prospective Cohort Study**

Yen-Fu Chen, Hsin-Han Hou, Ning Chien, Kai-Zen Lu, Ying-Yin Chen, Zheng-Ci Hung, Jung-Yien Chien, Hao-Chien Wang, Chong-Jen Yu

**Supplementary material**

| **Table S1.** The correlations between Type 2 biomarkers, inflammatory markers, and immunological profiles with the severity scores in bronchiectasis | | | | | | | |
| --- | --- | --- | --- | --- | --- | --- | --- |
| **Spearman correlation test** | BSI score | P value | E-FACED | P value | modified  Reiff score | P value |  |
| BEC | 0.093 | 0.294 | 0.190 | 0.030 | 0.222 | 0.011 |  |
| FeNO | -0.125 | 0.156 | -0.055 | 0.532 | -0.181 | 0.040 |  |
| Serum total IgE | 0.087 | 0.324 | 0.103 | 0.244 | 0.074 | 0.404 |  |
| Neutrophil-lymphocyte ratio | 0.304 | <0.001 | 0.309 | <0.001 | 0.260 | 0.003 |  |
| Neutrophils | 0.240 | 0.006 | 0.256 | 0.003 | 0.226 | 0.006 |  |
| Lymphocyte | -0.146 | 0.098 | -0.122 | 0.165 | -0.068 | 0.443 |  |
| BAL IL-1β | 0.392 | <0.001 | 0.309 | <0.001 | 0.539 | <0.001 |  |
| BAL IL-8 | 0.425 | <0.001 | 0.370 | <0.001 | 0.547 | <0.001 |  |
| BAL TNF-α | 0.340 | <0.001 | 0.279 | 0.001 | 0.481 | <0.001 |  |
| BAL IL-6 | 0.317 | <0.001 | 0.263 | 0.007 | 0.403 | <0.001 |  |
| BAL MCP-1 | 0.236 | 0.007 | 0.187 | 0.033 | 0.261 | 0.003 |  |
| CRP | 0.492 | <0.001 | 0.462 | <0.001 | 0.317 | <0.001 |  |

Spearman’s test assessed correlations between quantitative variables. BAL=Bronchoalveolar lavage; BEC= Blood eosinophil counts; BSI=Bronchiectasis severity index; CRP=C reactive protein; E-FACED=Exacerbation, forced expiratory volume in 1 second (FEV_1_), age, chronic colonization by *Pseudomonas aeruginos*a, radiological extension and dyspnea; FeNO=fractional exhaled nitric oxide, IgE=Immunoglobulin E; IL-1β=interleukin [IL]-1beta; IL-6=interleukin[IL]-6; IL-8=interleukin [IL]-8; MCP-1=Monocyte chemoattractant protein-1; NLR=Neutrophil-Lymphocyte ratio; TNF-α=tumor necrosis factor-alpha.

**Supplementary Figures**

**Figure S1. (**A-C) The correlation between BAL eosinophils (%) and BEC, FeNO and serum total IgE in the bronchiectasis cohort. BAL=Bronchoalveolar lavage; BEC=Blood eosinophil counts; FeNO=fractional exhaled nitric oxide; IgE=Immunoglobulin E.


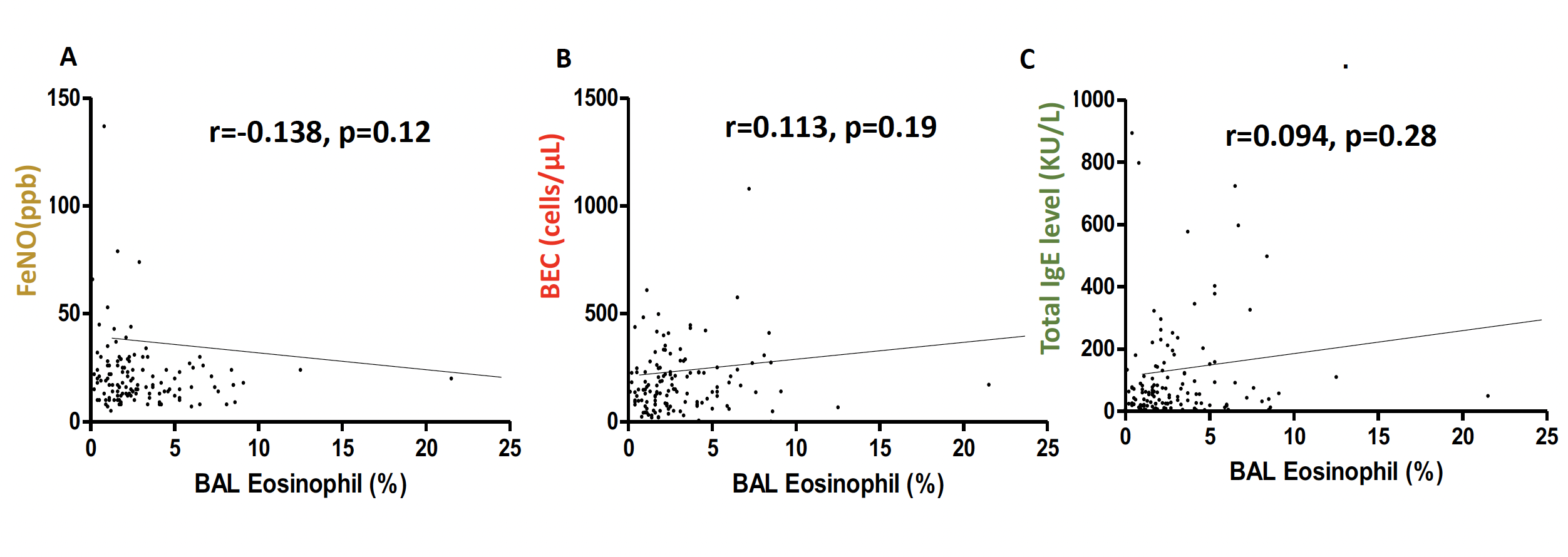


**Figure S2.** Kaplan-Meier curves illustrating the risk of future exacerbations in bronchiectasis patients, categorized by key clinical indicators: (A) Pseudomonas aeruginosa colonization, (B) Spirometry FEV1/FVC ratio (< 0.7 and > 0.7), (C) Neutrophil-to-lymphocyte ratio (NLR <3.0 and > 3.0), and (D) Bronchiectasis Severity Index (BSI) scores, with ranges indicating mild (0-4), moderate (5-8), and severe (>9) disease. Each factor is depicted in relation to its impact on exacerbation risk. FEV1=Forced expiratory volume in 1 second, FVC=Forced vital capacity.


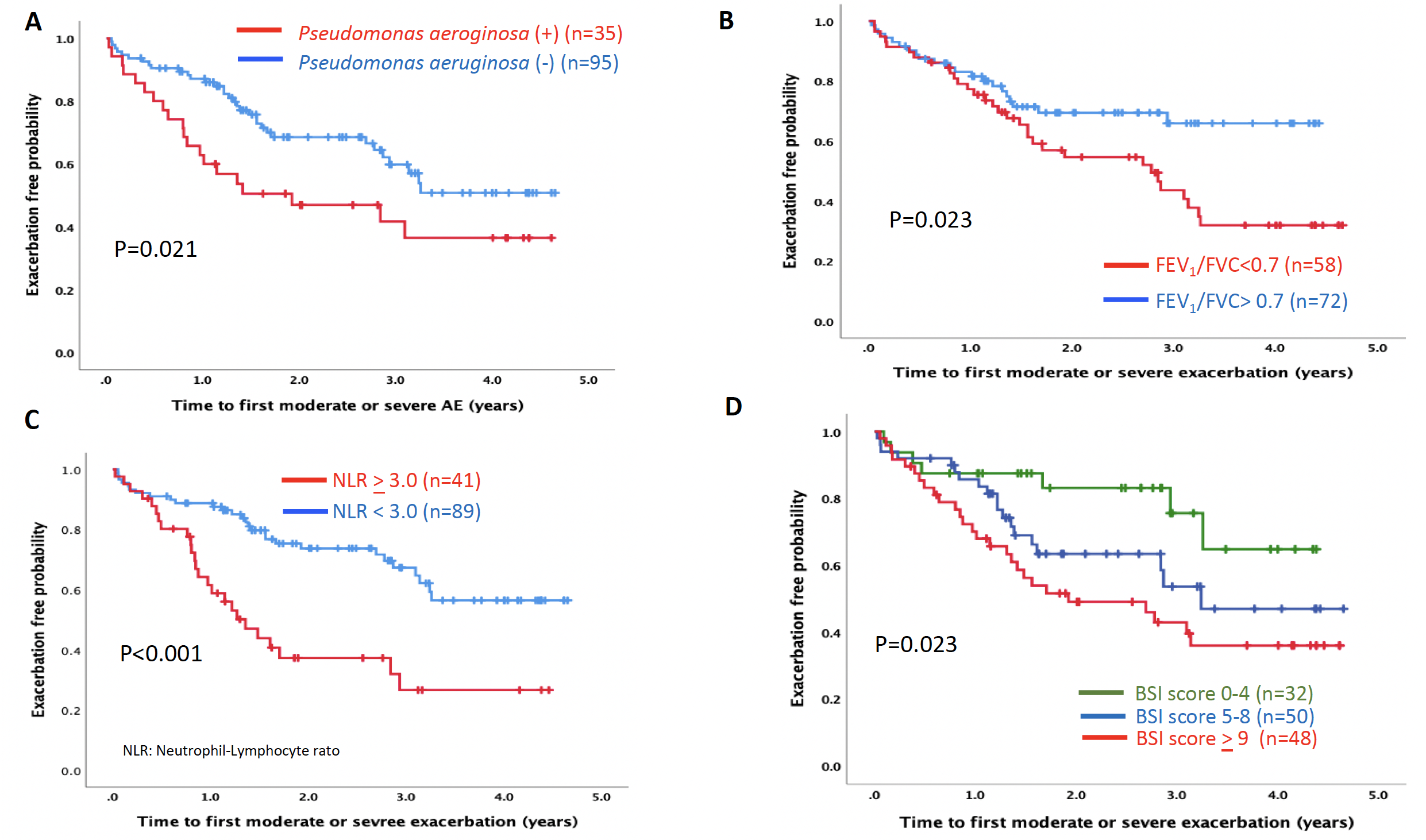


**Figure S3**: Kaplan-Meier curves illustrating the risk of future exacerbations in bronchiectasis patients, categorized by key clinical factors and management approaches: (A) modified Medical Research Council (mMRC) dyspnea scale scores (ranging from 0-1 and 2-4), (B) exposure to inhalation therapy (including monotherapy, dual bronchodilator and triple therapy), (C) use of inhaled corticosteroids (ICS), and (D) previous exposure to macrolides. Each curve depicts the specific impact of these factors on the risk of exacerbation.


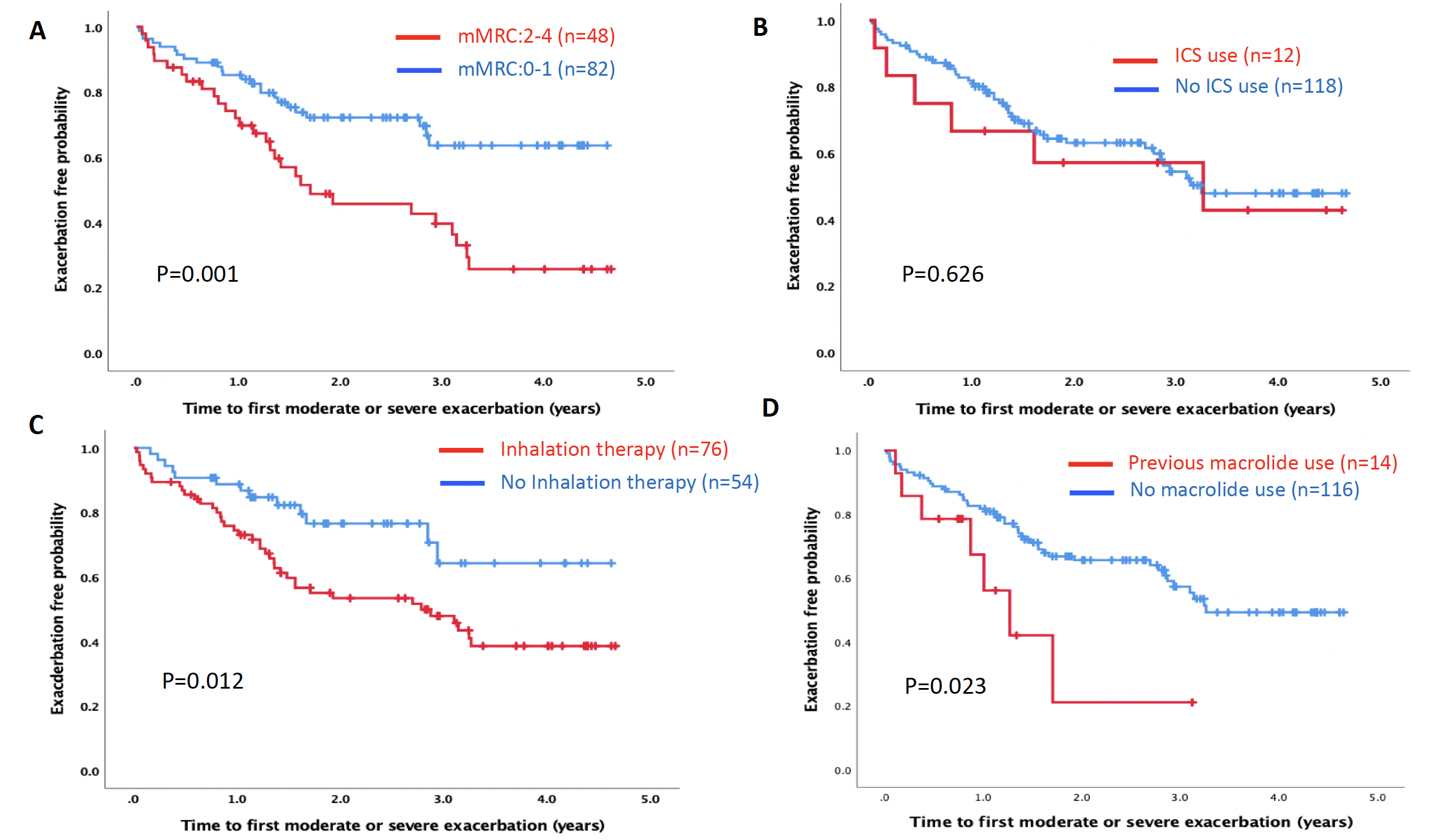

Supplement: Supplementary file 1 — Supplementary file1 (DOCX 8103 KB) [file 408_2024_707_MOESM1_ESM.docx]
